# Evaluating clinician experience in value-based health care: the development and validation of the Clinician Experience Measure (CEM)

**DOI:** 10.1186/s12913-022-08900-8

**Published:** 2022-12-06

**Authors:** Reema Harrison, Elizabeth Manias, Louise Ellis, Laurel Mimmo, Ramesh Walpola, Ben Roxas-Harris, Timothy Dobbins, Rebecca Mitchell, Sharyn Cowie, Glen Maberly, Catherine Chan, Liz Hay

**Affiliations:** 1 Centre for Health Systems and Safety, Australian Institute of Health Innovation, Macquarie University, Lvl 6, 75 Talavera Road, Sydney, NSW 2109 Australia; 2 Faculty of Medicine, Nursing and Health Sciences, School of Nursing and Midwifery, Monash University, Clayton Campus, Wellington Road, Clayton, Victoria 3800 Australia; 3 Deakin University, Burwood, Australia; 4 Centre for Healthcare Resilience and Implementation Science, Australian Institute of Health Innovation, Macquarie University, Sydney, NSW 2109 Australia; 5 School of Women’s and Children’s Health, UNSW, Sydney, 2052 Australia; 6 School of Population Health, UNSW Sydney, Sydney, 2052 Australia; 7 Australian Institute of Health Innovation, Macquarie University, Sydney, NSW 2109 Australia; 8 Western NSW Local Health District, Broken Hill, NSW 2880 Australia; 9 Blacktown and Western Sydney Local Health District, North Parramatta, NSW 2151 Australia; 10 Strategic Reform Branch, NSW Ministry of Health, Sydney, NSW 2065 Australia

**Keywords:** Clinician experience measurement, Value-based healthcare, Health services, Survey, Psychometrics, Validity, Reliability

## Abstract

**Background:**

Clinicians’ experiences of providing care constitute an important outcome for evaluating care from a value-based healthcare perspective. Yet no currently available instruments have been designed and validated for assessing clinicians’ experiences. This research sought to address this important gap by developing and validating a novel instrument in a public health system in Australia.

**Methods:**

A multi-method project was conducted using co-design with 12 clinician leaders from a range of NSW Health Local Health Districts to develop the Clinician Experience Measure (CEM). Validity and reliability analyses were conducted in two stages, first assessing face and content validity with a pool of 25 clinicians and then using psychometric analysis with data from 433 clinicians, including nurses, doctors and allied health and representing all districts within one jurisdiction in Australia.

**Results:**

Data gathered from 25 clinicians via the face and content validity process indicated that the initial 31-items were relevant to the range of staff employed in the NSW state health system, with minor edits made to the survey layout and wording within two items. Psychometric analysis led to a rationalised 18-item final instrument, comprising four domains: psychological safety (4-items); quality of care (5-items); clinician engagement (4-items) and interprofessional collaboration (5-items). The 18-item four-factor model produced a good fit to the data and high levels of reliability, with factor loadings ranging from .62 to .94, with Cronbach’s alpha (range: .83 to .96) and composite reliability (range: .85 to .97).

**Conclusions:**

The CEM is an instrument to capture clinicians’ experiences of providing care across a health system. The CEM provides a useful tool for healthcare leaders and policy makers to benchmark and assess the impact of value-based care initiatives and direct change efforts.

## Background

Value based health care (VBHC) takes a whole-of-health system focus, aiming to address health outcomes that matter to patients relative to the resources or costs of care provision when considered over a full cycle of care [1]. Value-based health care (VBHC) includes consideration of aspects of healthcare delivery that impact individual’s experiences, clinical, effectiveness and efficiency outcomes. In NSW where the current study was located, value-based approaches are considered to create benefits to system stakeholders at all levels by improving health outcomes that matter to patients, the experiences of receiving care, the experiences of providing care, and the effectiveness and efficiency of care. A focus on value-based outcomes has necessitated a shift away from system and service performance that centre on the volume of activity. To measure the outcomes of VBHC approaches, healthcare services consider measures relating to patient-reported experience and care outcomes, clinical and patient reported quality of life outcomes, efficiency data, and data about clinician experiences of providing care [2, 3].

Health systems internationally have addressed VBHC through three major approaches, with notable variations between different systems. Firstly, by seeking to pivot away from traditional financial models which focus on fee for service or capitation and establishing financial models based on achieving better clinical outcomes [4]. Secondly, by evaluating routinely captured data on patient-reported outcomes measures (PROMs) and patient-reported experience measures (PREMs) [5, 6]. Thirdly, by placing greater emphasis on efficiency of care and reducing waste [7, 8]. In contrast, measurement and evaluation of system performance based on clinicians’ experiences of providing care has received limited attention internationally. Lack of outcome measures to determine clinicians’ experiences in the context of VBHC limit the ability of health systems and researchers to evaluate the effects of VBHC programs and initiatives.

As key actors in health systems and services, clinicians from all professions have vital roles in promoting health through the planning and delivery of care. Clinicians’ experiences of providing care, including their ability to input into decision-making, of providing the safest possible care, and of being respected and valued by colleagues, have been associated with both better clinician well-being and patient outcomes [9]. Conversely, detrimental experiences, such as emotional exhaustion due to work pressures, have been associated with poorer clinical outcomes [10]. The influential role of teamwork and relationships with team members in contributing to, and in mitigating the effects of, negative workplace experiences on clinicians have also been widely documented [11, 12]. In recognising that clinicians’ experiences influence healthcare outcomes, including the quality and safety of care, health systems are seeking to monitor and optimise clinicians’ experiences of providing care to improve health system function [3].

Despite its importance, clinician experience is a poorly defined concept, creating challenges for health systems to embed routine measurement and improvement of clinician experience [9]. In a review of published and grey literature, we established that no existing measurement instruments assess clinician experiences of providing care that could be applied system-wide [9]. Several included studies did however report qualitative and survey data about clinicians’ experiences of specific events. From synthesising this evidence, we identified common factors that were central to a clinicians’ experience of providing care and using this evidence, we defined ‘clinician experience’ as *clinicians’ perceptions of the quality and safety of care provision, interprofessional collaboration and work environment, their engagement in decision-making and psychological experiences in the workplace* [9]. For health systems to benchmark and assess the success of initiatives to assess and improve clinicians’ experiences, a set of common measures to capture clinicians’ experiences of providing care system-wide is required.

Developing a measure of clinician experience contributes to New South Wales (NSW) Health’s strategic priority of delivering value-based healthcare and NSW’s strategic vision of delivering value-based healthcare. New South Wales is one of the eight states and territories within Australia, each state holds responsibility for state-wide healthcare services under a wider federal Department of Health. A key priority of the federal Department of Health is to achieve optimal healthcare outcomes and experiences at the lowest cost; characterised as value-based health care. To address this gap, our research aimed to collaboratively develop and validate a clinician experience measure in a state-wide public health system. The purpose of this article is to report the process of developing and conducting initial validation work to create a Clinician Experience Measure that is suitable for use to evaluate value-based care projects in the Australian health system.

## Method

### Design

A multi-method approach was employed in a sequential study using co-design workshops to develop the Clinician Experience Measure (CEM) and a cross-sectional survey to provide data with which to undertake preliminary validation of the instrument. Specifically, to create this new measure, we took the following approach:

1. Co-design workshops with clinician leaders to identify key components of clinician experience, in the context of published literature and currently available measurement instruments of staff experiences in healthcare.
2. Cognitive interviewing with health professionals to refine the proposed survey items.
3. Online survey of the proposed instrument for further item refinement and assessment of its construct validity and internal consistency.

#### Phase 1: co-design workshops

##### Sampling and setting

Clinicians who were nurses, doctors, pharmacists, and allied health staff from a public health system in one Australian state (NSW) were eligible to take part. For the co-design workshops, clinician leaders from NSW Health were invited to participate in one of two workshops, from each of the NSW local health districts and speciality networks (e.g. paediatrics) across metropolitan, regional and rural areas across the state, and from all service areas. Invitations to contribute to the co-design were distributed via email to clinical leads across the states 30 communities of practice by the Ministry of Health, with an option to opt in to one of the range of group meeting times provided. Those who wished to contribute were able to indicate which is the times they were able to join, and these times were scheduled at different times of day and for different days of the week over a two-week period to ensure a range of clinicians could take part. Nineteen clinician leaders opted to contribute, with 12 clinicians ultimately attending the sessions comprising: 6 doctors; 4 allied health professionals and 2 nurses.

##### Procedure

Two, 90-minute workshops were held with 12 clinicians who responded to an invitation for co-design members (9 male and three female) to assist with developing a CEM. Members represented nine local health districts and specialty networks within the participating public health system. Online video-conferencing software was used to conduct the workshops due to COVID-19 restrictions. Both workshops were facilitated by the lead author (RH). Prior to the workshops, members were provided with a copy of a literature review reporting current evidence about the measurement of clinician experience of providing care, along with key discussion items. In the first workshop, the group initially discussed the key components that comprise clinician experience from their own perspective, generating a synergy of ideas that were then discussed in the context of the literature review evidence and currently available measurement instruments of staff experiences in healthcare that may be relevant to assess the emerging components of clinician experience [13–15].

In the second workshop, the research team presented to members a range of existing scales that measure concepts relating to clinician experience discussed in workshop one. These scales were identified from the research literature as those which had shown strong reliability. Members discussed the perceived relevance, feasibility in terms of length, and message framing of the existing scales for assessing clinician experience of providing care. The workshop members determined the existing scales they preferred based on their relevance, feasibility and framing relevant to the project aims, and the concepts for which novel items or scales were required. Throughout the process, workshop members reflected, through ongoing discussion and debate, on concepts of clinician experience that were critical for inclusion in a measurement instrument, those considered relevant but not critical for system-wide measurement, and those considered not relevant. At the conclusion of each workshop, the facilitator summarised the discussion.

After two workshops had been conducted, the clinician preferred scales and items identified from each workshop were consolidated into a prototype instrument by the workshop facilitator. The resulting prototype was then disseminated to the workshop members for further feedback and refinement before being finalised into a 31-item online instrument – the Clinician Experience Measure (CEM)- which was uploaded within Qualtrics for administration across the state-wide health system for the purposes of validation. The 31-item measure comprised of five domains: Quality of Care (9 items on a 7-point Likert scale); Psychological Safety (7 items on a 7-point Likert scale); Confidence/Self-Efficacy (1 item on an 7-point Likert scale); Interprofessional Collaboration (8 items on a 7-point Likert scale); and Clinician Engagement (6 items on a 5-point Likert scale).

#### Phase 2: preliminary testing and initial validity analyses

Face validity analyses were completed ahead of administering the instrument by circulating the prototype instrument to 25 clinicians across the health system from a range of professions.

##### Sampling

The instrument was distributed to clinical leads through the health system’s 30 communities of practice to reach a diverse range of localities and services in metropolitan, regional and rural areas. Clinicians were invited to review each item in an online version of the instrument and to provide written anonymous feedback in free-text boxes below each item about whether they perceived the content was relevant to clinician experience, whether fundamental content was missing and ease of understanding. The online survey was open for the capture of this preliminary validity data for 2 weeks and 25 clinicians from rural, regional, and metropolitan services responded. The final sample that reviewed the initial instrument comprised of 7 doctors, 9 nurses, 5 allied health staff, and four clinician managers. This process indicated that the instrument reflected fundamental content about the major contributors to clinician experiences from NSW Health. The instrument then was then distributed to a larger group > 500 clinicians for reliability analysis in phase 3.

#### Phase 3: CEM refinement and reliability analyses

##### Sampling and procedure

An embedded link to the CEM was distributed through the health system’s 30 communities of practice and was able to be completed anonymously by clinicians. The link remained active for a three-week period during October 2020, with one reminder sent. Once the link was deactivated, the project team downloaded the data from the Qualtrics platform for analysis. The resulting data were used refine and reduce the 31-item instrument to produce an instrument that had strong psychometric qualities but would be short enough to be completed by busy clinicians.

Data from respondents for whom more than 10% of data were missing were excluded from the analyses. Remaining missing values were imputed using the Expectation Maximisation Algorithm within SPSS v26 [16, 17]. Two items related to psychological safety were reverse coded so that higher item-response scores indicated greater psychological safety. Frequency distributions were calculated to test whether items violated the assumption of univariate normality (i.e., skewness index ≥3, kurtosis index ≥10) [18]. An exploratory factor analysis was performed on the draft 31-item set to determine the number of underlying factors and to undertake any necessary initial item reduction. Principal axis factoring with oblique rotation was performed, retaining factors of eigenvalues > 1. An item was retained if it loaded > 0.6 on its primary factor. Items that had cross-loading or loadings < 0.6 were eliminated.

A commonly used method to investigate construct validity is confirmatory factor analysis (CFA). Like exploratory factor analysis (EFA), CFA is a statistical method used to reduce the overall number of observed variable into latent factors based on commonalities within the data. However, CFA assists in the reduction of measurement error and allows for the testing of an a priori model at the latent factor level [19]. Here, the retained items were evaluated psychometrically via confirmatory factor analysis (CFA), using a two-stage process. First, one-factor congeneric models were run using AMOS, version 25 [10]. The analytic plan involved removing one item at a time from each model using the following strategy: (i) removing items through inspection of factor loadings, modification indices, and item content; (ii) removing items if each construct contained at least four observed variables; and (iii) removing items if the resulting model demonstrated an improved model fit [11, 12]. Second, the full-factor model was run with the reduced item set. Each item was loaded on the one factor it purported to represent.

Further item refinement was undertaken as required through inspection of factor loadings and modification indices. Goodness-of-fit was assessed using the Tucker Lewis Index (TLI), Comparative Fit Index (CFI), Root Mean Square Error of Approximation (RMSEA) and the relative chi-square (chi-square/df). The TLI and CFI yield values ranging from zero to 1.00, with values greater than .90 and .95 being indicative of acceptable and excellent fit to the data [13]. For RMSEA, values less than .05 indicate good fit, and values as high as .08 represent reasonable errors of approximation in the population [14]. Chi-square tests are sensitive to sample size [15], therefore the relative chi-square (chi-square/df) was used as an index of fit, with values less than two indicating a good model fit [16]. Reliability of each of the subscales was assessed through split-half reliability, equivalent to Cronbach’s alpha (using SPSS v26), and composite reliability (using AMOS v26).

## Results

### Phase 1: co-design workshops

Data were gathered from 12 clinician leaders. The CEM comprised of three separate existing scales of 1) psychological safety (7-item measure) [20], 2) self-efficacy (single-item measure) [21] and 3) inter-professional cooperation (8-item subscale) [22]. Two novel measures were co-created through the workshops: a 9-item measure of experience regarding quality of care, based on the Donabedian and Institute of Medicine Quality of Care Models (Donabedian, 1962; IOM 2000). A further 6 items were co-developed to assess clinician engagement utilising the Victorian Clinician Engagement Framework [23]. Additional to the 31-item CEM, a series of demographic information items, such as professional role, years of experience, age and speciality were also co-developed through the workshop sessions relevant to the participating health system.

### Phase 2: face and content validity

Data gathered from 25 clinicians via the face and content validity process indicated that the included items were relevant to the range of staff employed in the state health system, with minor edits made to distinguish the set of items regarding individual characteristics from those relating to work role characteristics in the survey layout. This process revealed the tool to be of an acceptable duration for completion by busy clinical staff within 10 minutes. Qualitative comments received from clinicians made suggestions for a free-text item to be included to enquire how could experiences be improved and queried the use of reverse scored items in the domain of psychological safety. Comments received led to the wording of two items being edited for clarity. The reverse scored items were retained because they were part of an existing validated scale and free-text comments were not added to the core measure but remain optional additions for local teams to include where relevant to their activities.

### Phase 3: CEM item refinement and assessment of construct validity and internal consistency reliability analyses

Data were received from 433 clinician respondents of the 572 who received the instrument (75.7% response rate) across the range of specialty areas shown in Table 1. Respondents were predominantly female (*n* = 304; 70%) and aged between 30 and 60 years (*n* = 321; 73%). Many of the respondents had several years of experience, with more than half (*n* = 225; 52%) of respondents having been in their profession more than 10 years (Table 1).

**Table 1:** Demographic information of clinicians

Characteristic	*N* = 433^a^ (%)
Sex	
Female	304 (70)
Male	70 (16)
Other/Prefer not to respond	29 (6.7)
Missing	30 (6.9)
Age group	
21–30	38 (8.8)
31–40	110 (25)
41–50	101 (23)
51–60	110 (25)
61–70	34 (7.9)
Over 70	0 (0%)
Missing	40 (9.2)
Years in profession	
Less than 2 years	14 (3.2)
2–5 years	33 (7.6)
6–10 years	66 (15)
11–20 years	114 (26)
21–30 years	111 (26)
More than 30 years	0 (0)
Missing	95 (22)
Indigenous status	
Aboriginal descent	7 (1.6)
Neither Aboriginal nor Torres Strait Islander descent	356 (82)
Prefer not to respond	39 (9.0)
Missing	31 (7.2)
Manage other staff	
No	251 (58)
Yes	158 (36)
Missing	24 (5.5)
Professional Role	
Allied health professional	236 (54.5)
Clinician manager	35 (8.1)
Doctor / Surgeon	45 (10.4)
Nurse / Midwife	82 (18.9)
Paramedic	1 (0.2)
Pharmacist	11 (2.5)
Other (unspecified)	23 (5.3)
No response	23 (5.3)
Specialty Area	
Aged Care	48 (11.1)
Anaesthesia	3 (0.7)
Emergency Care / Emergency Department	10 (2.3)
Endocrinology	7 (1.6)
General medicine	25 (5.8)
General surgery	12 (2.8)
Intensive Care	9 (2.1)
Medical Imaging	1 (0.2)
Mental Health	79 (18.2)
Nephrology	9 (2.1)
Neurology	16 (3.7)
Oncology	20 (4.6)
Other (please specify)	134 (30.9)
Paediatrics child and youth	57 (13.2)
Pathology	3 (0.7)
Perioperative care	2 (0.5)
Rehabilitation care	53 (12.2)
Respiratory	10 (2.3)
No response	50 (11.5)

^a^Statistics presented: n (%)

Table 2 demonstrates descriptive statistics for each item within the CEM domains. As shown in the Table 2, none of the items within the CEM domains violated the established criteria of skewness and kurtosis. Asterisked items were ultimately removed from the CEM based on EFA and CFA analyses. EFA identified that there were four factors with eigenvalues greater than one, explaining 72.9% of the variance. These four factors have been labelled: (i) psychological safety; (ii) quality of care; (iii) interprofessional collaboration; and (iv) clinician engagement. The self-efficacy item heavily loaded onto the quality-of-care factor and thus, did not emerge as a separate factor. For the purposes of this analysis, items were retained if their primary factor loading was > 0.5.

**Table 2:** Descriptive statistics for all clinician experience measure items

Factor	Item label	Item wording	Mean	SD	Skewness Index	Kurtosis Index
**Psychological safety**	SAFE1 (R)	If I make a mistake in this department/unit, it is held against me	5.13	1.788	−.870	−.275
	SAFE2	Members of staff in this department are able to bring up problems and tough issues	4.89	1.732	−.698	−.447
	SAFE3 (R)	Members of staff in this department/unit sometimes reject others for having different perspectives	4.74	1.943	−.492	−.998
	SAFE4	I feel safe to take a risk in this department/unit	4.27	1.754	−.354	−.772
	SAFE5 (R)	It is difficult to ask other members of this department/unit for help	5.43	1.720	−1.002	−.036
	SAFE6	Members of staff in this department/unit would not deliberately act in a way that undermines me	4.88	1.995	−.650	−.898
	SAFE7	In working with members of this department/unit, my unique skills and talents are valued and utilised	5.16	1.700	−.923	.039
Self-efficacy	EFFC1	Rate how confident you are that you can currently provide high quality patient care	5.71	1.237	−1.453	2.536
Interprofessional collaboration	IPC1	My colleagues respect and trust each other	5.29	1.541	−1.032	.577
	IPC2	My colleagues share decision-making power with each other	4.93	1.691	−.853	−.136
	IPC3	My colleagues are open and honest with each other	4.99	1.639	−.824	.013
	IPC4	My colleagues make changes to our working approaches based on each other’s feedback	4.89	1.677	−.749	−.173
	IPC5	My colleagues strive to achieve mutually satisfying resolutions to differences of opinion	4.89	1.670	−.716	−.227
	IPC6	My colleagues understand the boundaries of what each other can do	4.99	1.709	−.899	.020
	IPC7	My colleagues understand that there are shared knowledge and skills between healthcare professionals in the team	5.41	1.648	−1.187	.663
	IPC8	My colleagues establish a sense of trust amongst our team members	5.08	1.723	−.910	−.041
Quality of care	QOC1	I have the infrastructure (equipment, policy and process) I need to deliver care safely	5.12	1.54	−.947	.324
	QOC2	I have the training and professional development required to deliver care safely	5.82	1.19	−1.721	3.781
	QOC3	I am able to deliver care efficiently (with maximum productivity with minimum wasted effort)	4.80	1.541	−.698	−.155
	QOC4	I am able to be responsive to the needs of individual patients	5.44	1.349	−1.0089	.938
	QOC5	I am able to provide care in a timely manner	5.09	1.386	−.831	−.457
	QOC6	I am able to provide care aligned with the currently accepted best practice	5.45	1.414	−1.283	1.486
	QOC7	I am able to provide care that is accessible to all patient populations	4.79	1.603	−.698	−.215
	QOC8	I am able to provide care that leads to a positive patient experience	5.68	1.203	−1.32	2.222
	QOC9	I am able to provide care that delivers an equal or superior clinical outcome	5.40	1.351	−1.142	1.043
Clinician engagement	ENGE1	I have the skills and knowledge required to participate in decision-making	5.78	1279	−1.737	3.694
	ENGE2	I have the opportunity to participate in decision-making my organisation	4.62	1.800	−.624	−.577
	ENGE3	I have the capacity in my work arrangements to participate in decision-making	4.75	1.713	.715	−.349
	ENGE4	I have opportunities to collaborate in the process of making changes in my organisation	4.50	1.771	−.573	−.627
	ENGE5	My voice is heard in the process of making changes in my organisation	4.20	1.863	−.337	−1.002
	ENGE6	There are mechanisms for me to take part in decision-making in my organisation	4.40	1808	−.434	−.834

Note. Analyses based on *N* = 461. (R) Items reverse coded

To further refine the item pool, a series of one-factor congeneric models were run for items designed to measure psychological safety, interprofessional collaboration, quality of care and clinician engagement (Table 3). Based on an examination of the standardised factor loadings and modification indices, items were removed one at a time, until the strongest items remained. The reduced item constructs demonstrated improved model fit statistics relative to the full models with all items.

**Table 3:** Model fit for the one factor congeneric models

Construct	χ^**2**^	df	TLI^a^	CFI^a^	RMSEA^a^
Psychological safety					
All items (6 items)	75.81	9	.90	.94	.13
Reduced Model (4 items)	1.96	2	1.00	1.00	.00
Interprofessional cooperation					
All items (8 items)	188.07	20	.95	.97	.14
Reduced items (5 items)	8.58	5	1.00	1.00	.04
Quality of care					
All items (10 items)	243.69	35	.91	.93	.11
Reduced items (5 items)	28.48	5	0.96	0.98	.10
Clinician engagement					
All items (6 items)	104.98	9	.94	.97	.15
Reduced items (4 items)	33.24	2	0.96	0.99	.15

^a^Root Mean Square Error of Approximation (RMSEA), Comparative Fit Index (CFI), and Tucker-Lewis index (TLI)

The reduced 18-item four-factor model was then tested through CFA using the same sample. Each item was loaded on the one factor it purported to represent. The 18-item four-factor model produced a satisfactory fit to the data, χ^2^ (129) = 290.42, TLI = .98, CFI = .98, RMSEA = .05, with the self-efficacy item collapsed into the quality of care items. The factor loadings for each of the 18-items, ranged from .62 to .94 (*M* = .84). Cronbach’s alpha and composite reliability for the final items are shown in Table 4, demonstrating that all four factors demonstrated high levels of internal consistency. Correlations between the factors were significant but were generally low to moderate (range = .38 to .78), suggesting good discriminant validity between factors [24].

**Table 4:** Confirmatory factor analysis for reduced four factor model

Construct	Item label	Item	Factor loadings	Coefficient alpha	Composite reliability
Psychological safety	SAFE1*	If I make a mistake in this department/unit, it is held against me	.62	.83	.85
	SAFE2	Members of staff in this department are able to bring up problems and tough issues	.82		
	SAFE4	I feel safe to take a risk in this department/unit	.76		
	SAFE7	In working with members of this department/unit, my unique skills and talents are valued and utilised	.78		
Interprofessional collaboration	IPC2	My colleagues share decision-making power with each other	.91	.97	.97
	IPC3	My colleagues are open and honest with each other	.92		
	IPC4	My colleagues make changes to our working approaches based on each other’s feedback	.91		
	IPC5	My colleagues strive to achieve mutually satisfying resolutions to differences of opinion	.93		
	IPC8	My colleagues establish a sense of trust amongst our team members	.94		
Quality of care	QOC4	I am able to be responsive to the needs of individual patients	.84	.89	.90
	QOC5	I am able to provide care in a timely manner	.78		
	QOC6	I am able to provide care aligned with the currently accepted best practice	.83		
	QOC8	I am able to provide care that leads to a positive patient experience	.82		
	EFFC1	I am confident that I am able to provide high quality patient care	.63		
Clinician engagement	ENGE2	I have the opportunity to participate in decision-making in my organisation	.91	.96	.96
	ENGE4	I have opportunities to collaborate in the process of making changes in my organisation	.94		
	ENGE5	My voice is heard in the process of making changes in my organisation	.93		
	ENGE6	There are mechanisms for me to take part in decision-making about in my organisation	.93		

Note. * Items reverse coded

### Reliability

The 18-item four factor model was run separately on two randomly allocated sub-datasets, both producing satisfactory model fit. Data-set A (*n* = 230): χ^2^ (129) = 208.96, TLI = .98, CFI = .98, RMSEA = .05; Data-set B (*n* = 231): χ^2^ (129) = 232.85, TLI = .97, CFI = .97, RMSEA = .06 (∆ χ^2^ = 23.9; *p* > .05, NS).

The resulting final CEM instrument, following the co-production and validation process which has been documented, constituted four domains, contained 18-items and demonstrated strong psychometric qualities (Table 4). The 18-item four-factor model produced a good fit to the data, χ^2^ (129) = 290.42, TLI = .98, CFI = .98, RMSEA = .05, with factor loadings ranging from .62 to .94. Cronbach’s alpha (range: .83 to .96) and composite reliability (range: .83 to .97) demonstrated that all four factors had high levels of internal consistency.

## Discussion

The CEM comprises 18 items distributed across four domains of clinician experiences of providing care. The CEM is, to our knowledge, the first measure that has been developed and validated to capture clinician experience of providing care relevant to a value-based care framework. As an 18-item measure, the CEM provides a brief survey tool that can be used for routine system-wide benchmarking and to inform potential health system improvement activities. The CEM demonstrates strong internal consistency and can be applied to diverse discipline groups of clinicians to compare experiences amongst different cohorts and in a range of healthcare delivery settings. This novel measure may be further validated for its psychometric properties and across health settings nationally and internationally.

Our recent literature review demonstrated that, to date, clinician experiences have been commonly assessed in the context of specific initiatives, models of care, practices or events arising in healthcare, situated in a vast literature of clinicians’ psychological experiences in relation to their work [13]. Only two identified pieces of work sought to assess clinician experience more broadly, at a service- or system-wide level [14, 15]. The two identified surveys addressed clinician experience as part of the Picker staff survey adapted for healthcare staff in conjunction with, and in relation to, employment matters. As such, these surveys are lengthy (≥75 items) and extend beyond the scope of clinician experience in a value-based framework, for example with items assessing whether rosters are perceived the be fair or released with sufficient notice, how tired individuals feel when completing overtime and the perceived helpfulness of training [14]. Nonetheless, there are parallels in the key concerns of clinicians in the focus of both the co-designed CEM and existing staff surveys on inter-personal relationships and their implications for safety of patient care. Notable in the CEM validation work was the interrelationship between domains established through performing factor analysis. The relationship between domains highlights contemporary issues, such as the experience of psychological safety in the workplace, which impact not only clinician experience, but also clinician satisfaction within their work environment. Clinicians’ experiences captured system-wide via value-based care programs must be considered in the context of human resource management, which has been increasingly recognised as important for optimal health system function since the COVID-19 pandemic.

### Implications

Applications of the CEM in system-wide assessment as part of value-based care programs may provide a method for identifying trends between and within services, localities, and professional groups to evaluate or compare the impacts of value-based care interventions and initiatives. These data may provide indication of areas of concern or that warrant further analysis to direct health system or service efforts to address workforce and quality of care issues. The instrument may also be useful in workforce planning, or as an indicator of factors driving staff retention. The relatively short survey length allows for it to be easily implemented as part of value-based healthcare initiative assessments, enabling timely data gathering and analysis to be performed compared to the collation of lengthy staff surveys or instruments that rely on qualitative data. The CEM has further applications in research focused on health systems performance and change, enabling comparisons to be drawn between studies that utilise this as a common measure.

### Limitations

Whilst the CEM provides a valuable, novel, and validated measure to assess clinician experiences in a value-based care framework, there are notable potential selection biases that may have influenced the design and content of the resulting instrument. By co-developing this measure with a small group of clinicians from a single health system, the resulting measure may be influenced by their lived experiences but also by local meso- and macro-contextual factors. Workshop attendees were also provided with a literature review of the current measurement approaches available to gathered clinician experience prior to the workshops, which may have influenced the resulting instrument. Data for psychometric testing were obtained from a large group but also across a single public health system, respondents were predominantly female, allied health professionals were over-represented, and most respondents had many years of experience; these factors may have shaped the resulting data. This is only a first phase of validation and other psychometric qualities should be documented, in addition to external validation from a perspective of transcultural adaptation. The resulting measure may be assessed for its relevance to other health systems and contexts given rising interest in creating value-based care. We suggest, therefore, that the instrument requires more expansive testing and validation including in diverse geographical locations in further Australian states, internationally, and with clinicians from various specialities.

## Conclusion

The 18-item CEM provides a brief instrument that can be used for routine system-wide benchmarking in the context of value-based care activities and initiatives. The instrument has further potential applications in identifying and directing system and service improvement activities and for research purposes. Whilst the CEM demonstrates strong internal reliability and relevance to diverse groups of clinicians to compare experiences amongst different cohorts and in a range of healthcare delivery settings, further validation in a range of localities is required. This novel measure has demonstrated its applicability to the one public health system and may now be further validated across health settings internationally.

## Data Availability

The data that support the findings of this study are available from NSW Health, but restrictions apply to the availability of these data, which were used under license for the current study, and so are not publicly available. Data may be requested however from the authors upon reasonable request and with permission of NSW Health.
